# Racialized Economic Segregation and Disparities in the Risk of Stillbirth

**DOI:** 10.1007/s40615-025-02511-9

**Published:** 2025-07-09

**Authors:** Amal Rammah, Jasmine Drake, Iman Moussa, Kristina W. Whitworth, Howard Henderson, Juan Alvarez, Elaine Symanski

**Affiliations:** 1 Center for Precision Environmental Health, Baylor College of Medicine, Houston, TX USA; 2 Department of Administration of Justice, Texas Southern University, Houston, TX USA; 3 Section of Epidemiology and Population Sciences, Department of Medicine, Baylor College of Medicine, Houston, TX USA

**Keywords:** Stillbirth, Disparities, Segregation, Spatial social polarization

## Abstract

**Background:**

While there is growing evidence of the negative impact of neighborhood segregation on maternal and infant health, the evidence for stillbirth is limited.

**Methods:**

A retrospective cohort study of live births and stillbirths for 2007–2020 was conducted in greater Houston, TX, to examine the associations of racialized economic segregation, assessed using the Index of Concentrations at the Extremes (ICE), with stillbirth. Here, ICE contrasted census tract-level racial and economic privilege with disadvantage (i.e., high-income non-Hispanic White persons vs. low-income Persons of Color). Additionally, Oaxaca-Blinder decomposition methods were applied to examine the degree to which segregation and maternal risk factors explained observed racial disparities in stillbirth.

**Results:**

The prevalence of stillbirth was highest among non-Hispanic Black mothers (9.1/1000) compared with Hispanic mothers (4.7/1000), non-Hispanic White mothers (3.8/1000), and mothers whose race or ethnicity was unknown or classified as other (3.7/1000). In models adjusted for age, pre-pregnancy body mass index (BMI), smoking during pregnancy, and education, odds of stillbirth were reduced for non-Hispanic White mothers living in the most privileged neighborhoods (OR = 0.82, 95% CI 0.60, 1.12 for ICE tertile 3 vs. ICE tertile 1). In contrast, residing in more privileged neighborhoods did not confer the same benefit for mothers of other racial and ethnic groups. Based on the decomposition analysis, maternal age, education, pre-pregnancy BMI, smoking, and ICE explained 21.2% of the disparity in stillbirth between non-Hispanic Black and non-Hispanic White mothers; of which, having a high school education or less (12.3%), living in the most privileged neighborhoods (9.7%), and being obese (7.2%) contributed the most.

**Conclusions:**

Neighborhood privilege appears to confer benefits for White mothers but not for non-White mothers, potentially due to experiences of racial discrimination that offset economic advantages. While racialized economic segregation contributed modestly to Black-White disparities in stillbirth, much of the disparity remains unexplained by the factors examined. Future research should explore additional individual- and neighborhood-level contributors to these disparities while addressing inequities to reduce stillbirth risks across all populations.

## Introduction

Stillbirth, often defined as the death of a fetus prior to birth at 20 weeks of gestation or more, is a public health concern with considerable mental health and financial burdens on families [1]. In the USA, disparities in stillbirth persist with the prevalence among non-Hispanic Black mothers more than twice that of non-Hispanic White or Hispanic mothers [2]. Individual-level risk factors for stillbirth do not fully explain these disparities [3–6] and social determinants of health (SDOH), such as air pollution [7], heat exposure [8], or segregation [9, 10], may play a role. For SDOH, several complex and interrelated pathways have been posited to explain the role of segregation on disparities in adverse birth outcomes. Upstream factors include insufficient investment in local schools, health care facilities, housing, and businesses, which in turn can impact individual-level socioeconomic status, neighborhood quality, social capital, health behaviors, and exposure to stress [11]. Further, metrics that operationalize different aspects of segregation such as the degree to which a neighborhood has the same distribution of racial and ethnic groups as the region as a whole (evenness) or proxy measures like racial composition have been used in epidemiologic studies to evaluate associations with adverse birth outcomes; this heterogeneity in the assessment of segregation may contribute to equivocal findings [12].

While the number of studies of the impact of segregation on maternal and infant health outcomes is growing [12–15], the evidence for stillbirth is limited. The two earliest investigations in the 1950 s that examined vital statistics data for over 300 residential areas of New York City (with populations of about 25,000–30,000 persons in each area) reported higher prevalence of stillbirth among both Black and White mothers who lived in areas with larger proportions of non-White births [16, 17]. In a later ecologic investigation in Georgia relying on the dissimilarity index that calculated the proportion of Black individuals who would have to move between census tracts to be more evenly distributed across each county, the odds of stillbirth were highest among non-Hispanic Black mothers living in the most segregated counties (adjusted Odds Ratio (OR) = 1.15, 95% confidence interval (95% CI) 0.99, 1.33), whereas increased levels of segregation decreased risk of stillbirth for non-Hispanic White mothers (OR = 0.82, 95% CI: 0.71, 0.94) [18]. Another study that evaluated zip code tabulation area (ZCTA)-level measures of the dissimilarity index and the isolation index (that measures the probability of a member of one racial group interacting with a member of the same group), aggregated by Hospital Reference Region in the USA (*n* = 15), reported lower odds of stillbirth among both Black and White mothers living in less segregated regions of the USA [19]. For example, for the isolation index comparing mothers living in more integrated areas to more segregated areas, the adjusted OR for Black mothers was 0.25 (95% CI 0.16, 0.41), and for White mothers, it was 0.33 (95% CI 0.21, 0.53).

Given the focus of these prior studies on Black and White mothers, the impact of segregation on stillbirth among mothers of different racial or ethnic groups (e.g., Hispanic mothers) has not yet been examined. Additionally, the measures previously applied were one-dimensional measures of residential segregation, which oversimplify the complex interrelationships of social and economic factors, including historical policies and practices, such as redlining (a discriminatory practice of isolating racial or ethnic groups by restricting financial services or mortgage loans in certain disadvantaged areas) [20]. Further, they fail to fully account for how segregation reinforces economic inequality and concentrates poverty for minoritized communities, ultimately contributing to health disparities [21]. Finally, metrics employed in previous studies were constructed for relatively large geographic areas. In contrast, the Index of Concentration at the Extremes of race and income (ICE) assesses the joint impact of economic and racial and ethnic segregation and can be constructed at a relatively small geographic area (e.g., a census tract that has approximately 4000 residents) where segregation is likely to have a greater impact on health [22].

Owing to the paucity of evidence, we investigated the role of racialized economic segregation in explaining disparities in stillbirth in a large urban area with a diverse population, i.e., greater Houston, TX. Our first objective was to examine associations between segregation at the census tract-level and stillbirth and to evaluate the potential differential impact of living in segregated neighborhoods on stillbirth among different racial and ethnic groups. Given that little is known about what might explain racial and/or ethnic differences in stillbirth, we also examined the degree to which segregation and other risk factors contributed to these disparities. For this objective, we applied Oaxaca-Blinder decomposition, an innovative approach that is counterfactual in design, and by applying the prevalence of factors in one group to a second group, this method isolates the contribution of each factor in explaining the disparity in an outcome of interest between the two groups [23].

## Methods

### Study Population

We obtained 938,166 birth records for singleton live births in Harris County, TX (where Houston is located) with no congenital anomalies from the Texas Department of State Health Services (TX DSHS) from 2007 to 2020. By Texas statute, fetal death certificates are required for stillbirths, defined as an intrauterine death occurring at 20 weeks of gestation or later or, if the gestational age is unknown, at a weight of 350 g or more [24, 25]. Over the same time period and geographic area as the live birth records, we obtained 5750 fetal death certificates for stillbirths. For birth and fetal death certificates, the following information was provided by DSHS: geocoded address of residence at the time of delivery or fetal death, gestational age (weeks), maternal age (< 20, 20–24, 25–29, 30–34, ≥ 35 years), race and ethnicity (non-Hispanic White, non-Hispanic Black, Hispanic, Other/Unknown), nativity (US born, foreign born), education (high school or less, some college, bachelor’s degree or more), marital status (yes, no), fetal sex (male, female), gestational diabetes (yes, no), hypertensive complications of pregnancy (yes, no), and information about a prior pregnancy with a poor outcome (perinatal death or a newborn classified as small for gestational age (SGA) or with intrauterine growth restriction (IUGR)). Additionally, variables representing smoking during each trimester of pregnancy were used to create a dichotomous variable for smoking during pregnancy (yes, no). We also obtained mother’s height and pre-pregnancy weight, which were used to compute pre-pregnancy body mass index (BMI) (kg/m^2^), which was categorized as: < 18.5 (underweight), 18.5–24.9 (normal weight), 25.0–29.9 (overweight), and ≥ 30.0 (obese).

We excluded records for which the address was not geocoded (1.0%), was outside Harris County (0.8%), or assigned to census tracts with small population sizes (e.g., close to an airport) or for which the ICE metric could not be computed (0.003%). For live births, we also excluded records that had missing information on gestational age (0.02%), had gestational age less than 20 or greater than 44 completed weeks of gestation (0.06%), or had implausible combinations of gestational age and birth weight (0.07%) [26]. Because the live birth records data that was received from TX DSHS excluded infants born with congenital anomalies, we also excluded stillbirth records that indicated congenital anomalies as the cause of death (0.11%). Following these exclusions, 919,559 live births and 4834 stillbirths remained for analysis.

### Neighborhood-Level Racialized Economic Segregation

We determined the 20th and 80th percentiles of household income for census tracts in Harris County from the 2019 (5 years, 2015–2019) American Community Survey (ACS) (i.e., < $37,266 and > $90,052, respectively). Because the ACS estimates the proportion of individuals by race with household income in pre-defined categories, we constructed the ICE metric by defining the most privileged group as the proportion of non-Hispanic White persons with incomes > $75,000 (because the 80th percentile of household income in Harris County [$90,052] fell in the middle of the $75,000–$99,999 income bracket) and the most deprived group as the proportion of Persons of Color (POC) with incomes < $39,999 (because the 20th percentile of household income in Harris County [$37,266] fell in the $35,000 to $39,999 income bracket). The ICE metric ranges from − 1 (areas comprised solely of low-income POC, i.e., extreme deprivation) to 1 (areas comprised solely of high-income non-Hispanic White persons, i.e., extreme privilege). ICE scores were categorized into tertiles based on distributions in the study population, with tertiles 1 and 3 representing the most deprived and most privileged areas, respectively.

#### Statistical Analysis

To examine associations between ICE and stillbirth, we applied logistic regression models with generalized estimating equations to account for correlation among mothers residing in the same census tract and report ORs and 95% CIs. We identified the following maternal risk factors a priori to include in adjusted models: age, race and ethnicity, education, smoking during pregnancy, and pre-pregnancy BMI. Using a 10% change-in-estimate criterion [27], we additionally evaluated the potential for confounding from other maternal and fetal characteristics (i.e., nativity, marital status, gestational diabetes, hypertensive complications of pregnancy, history of previous pregnancy with a poor outcome and fetal sex). However, none of these variables met this criterion and were not included in the final models.

We examined the relative contributions of ICE, age, education, smoking during pregnancy, and pre-pregnancy BMI to observed racial and ethnic disparities in stillbirth using Blinder-Oaxaca decomposition methods [28] (specifying the cluster option in the Oaxaca command to account for the lack of independence in the outcomes among women living in the same census tract). These methods partition the gap in the prevalence or risk of an outcome between two groups into explained and unexplained portions. This is done in a counterfactual framework from the perspective of more disadvantaged mothers, and in this study, our focus was on the component that was explained by the differences in levels of each predictor in the model (i.e., the endowment effects). All statistical analyses were conducted in SAS, version 9.4 (Cary, NC, USA) except for the Oaxaca-Blinder decomposition that was conducted in Stata SE, version 16.1 (StataCorp LP, College Station, TX, USA). Statistical significance was assessed at *p* < 0.05.

## Results

As shown in Table 1, the prevalence of stillbirth was highest among non-Hispanic Black mothers (9.1/1000) as compared with Hispanic mothers (4.7/1000), non-Hispanic White mothers (3.8/1000), and mothers whose race or ethnicity was unknown or classified as other (3.7/1000). Most mothers were aged 25 to 34 years (53%), US born (60%), Hispanic (51%), married (55%), had a high school education or less (51%), were overweight or obese (pre-pregnancy BMI > 25 kg/m^2^) (51%), and reported not smoking during pregnancy (98%). Some differences in the sociodemographic profiles of mothers by race and ethnicity emerged. Whereas 70% of Hispanic mothers had a high school diploma or less, the percentages with this level of education were lower for all other groups (non-Hispanic Black mothers, 44%; mothers of unknown or “other” race and ethnicity, 23%; and non-Hispanic White mothers, 22%). The proportions of mothers that were overweight or obese were similar for non-Hispanic Black (58%) and Hispanic (57%) mothers and lower for non-Hispanic White mothers (41%) and mothers of other or unknown race or ethnicity (29%).

**Table 1:** Selected sociodemographic characteristics of mothers with live births or stillbirths by maternal race and ethnicity, Harris County, TX, 2007–2020

	All mothers *n* = 924,362	Non-Hispanic White mothers *n* = 209,055	Non-Hispanic Black mothers *n* = 168,777	Hispanic mothers *n* = 472,507	Mothers of other or unknown race or ethnicity *n* = 74,023
	%	%	%	%	%
**Still birth**					
Yes	0.52	0.38	0.91	0.47	0.37
**Mother’s age**					
< 20	8.92	4.07	10.41	11.64	1.91
20–24	22.76	15.14	28.04	26.29	9.71
25–29	27.91	29.1	28.11	27.43	27.21
30–34	25.07	33.06	20.92	21.12	37.23
≥ 35	15.32	18.63	12.46	13.52	23.93
Missing	0.02	0.01	0.06	0.01	0.01
**Mother’s education**					
High school or less	50.6	21.64	44.25	70.04	22.81
Some college	24.76	27.6	35.63	20.37	19.91
Bachelor’s degree or more	24.45	50.6	19.92	9.44	56.68
Missing	0.2	0.17	0.19	0.14	0.6
**Nativity**					
US-born	60.25	89.96	86.56	43.72	21.84
Foreign born	39.63	9.98	13.28	56.17	77.92
Missing	0.12	0.05	0.16	0.11	0.24
**Married**					
No	44.61	23.6	65.84	51.17	13.72
Yes	55.35	76.37	34.12	48.81	86.21
Missing	0.03	0.03	0.04	0.02	0.07
**Smoking during pregnancy**					
No	98.11	95.3	97.32	99.44	99.33
Yes	1.83	4.67	2.61	0.5	0.6
Missing	0.06	0.04	0.07	0.02	0.07
**Gestational diabetes**					
No	94.99	96.36	96.34	94.28	92.59
Yes	4.99	3.61	3.65	5.71	7.37
Missing	0.02	0.03	0.02	0	0.04
**Pre-pregnancy BMI**					
Underweight	3.75	4.26	3.47	2.85	8.7
Normal weight	43.86	53.25	37.1	39.53	60.39
Overweight	26.94	22.58	26.8	30.02	19.86
Obese	24.36	18.69	31.04	26.81	9.51
Missing	1.09	1.22	1.59	0.79	1.54
**Hypertensive disorders**					
No	94.7	94.54	93.02	95.06	96.68
Yes	5.28	5.41	6.95	4.93	3.27
Missing	0.03	0.05	0.04	0.01	0.05
**Previous pregnancy with a poor outcome**^**a**^					
No	98.6	98.96	97.91	98.66	98.8
Yes	1.38	1.01	2.08	1.33	1.16
Missing	0.02	0.04	0.02	0.01	0.04
**Fetal sex**					
Male	51.03	51.19	50.87	51.00	51.12
Female	48.97	48.81	49.13	49.00	48.87
**ICE**^**b**^					
Tertile 1	25.16	4.76	36.84	31.97	12.64
Tertile 2	24.67	9.63	29.47	30.34	20.08
Tertile 3	50.17	85.62	33.7	37.69	67.28

^a^Previous diagnosis of perinatal death/small-for-gestational age/intrauterine growth restriction

^b^Index of Concentration at the Extremes, contrasting high-income non-Hispanic White persons with low-income Persons of Color; tertile 1, most deprived; tertile 3, most privileged

In a fully adjusted model (Table 2), we observed positive, but statistically insignificant, ORs for ICE (OR = 1.02, 95% CI 0.94, 1.12 for tertile 2 vs. tertile 1 and OR = 1.02, 95% CI 0.94, 1.11 for tertile 3 vs. tertile 1). The largest disparities in stillbirth were observed for non-Hispanic Black mothers vs. non-Hispanic White mothers (OR = 2.02, 95% CI 1.82, 2.24). ORs were also increased for high pre-pregnancy BMI (OR = 1.84, 95% CI 1.70, 1.98, comparing obese mothers to those with normal weight) and older maternal age (OR = 1.57, 95% CI 1.43, 1.72 for 35 years and older vs. 25–29 years) and smoking (OR = 2.01, 95% CI 1.68, 2.39).

**Table 2:** Adjusted odds ratios (OR) and 95% confidence intervals (CI) for associations between Index of Concentration at the Extremes (ICE)^a^ and maternal risk factors and stillbirth, Harris County, TX, 2007–2020

	*n*^b^	OR (95% CI)
**ICE**		
Tertile 1	1170	Ref
Tertile 2	1114	1.02 (0.94, 1.12)
Tertile 3	1915	1.02 (0.94, 1.11)
**Race**		
Non-Hispanic White	717	Ref
Non-Hispanic Black	1267	2.02 (1.82, 2.24)
Hispanic	1984	1.11 (1.00, 1.23)
Unknown/other	231	1.01 (0.87, 1.18)
**Mother’s age (y)**		
< 20	373	1.10 (0.97, 1.25)
20–24	904	0.99 (0.91, 1.08)
25–29	1063	Ref
30–34	994	1.12 (1.02, 1.22)
≥ 35	865	1.57 (1.43, 1.72)
**Mother’s education**		
High school or less	2277	1.26 (1.14, 1.40)
Some college	1089	1.13 (1.02, 1.25)
Bachelor’s degree or more	833	Ref
**Pre-pregnancy BMI**		
Underweight	97	0.82 (0.66, 1.01)
Normal weight	1379	Ref
Overweight	1170	1.31 (1.20, 1.42)
Obese	1553	1.84 (1.70, 1.98)
**Smoking**		
Yes	155	2.01 (1.68, 2.39)
No	4044	Ref

^a^Contrasting high-income non-Hispanic White persons with low-income Persons of Color; tertile 1, most deprived; tertile 3, most privileged

^b^No. of stillbirths

The results from race and ethnicity-stratified analyses of the ICE-stillbirth association are shown in Table 3. For non-Hispanic White mothers, the odds of stillbirth were lower among mothers who lived in more privileged areas relative to the most deprived neighborhoods (OR = 0.85, 95% CI 0.60, 1.22; OR = 0.82, 95% CI 0.60, 1.12). For non-Hispanic Black and Hispanic mothers, there was little evidence of an association between ICE and stillbirth. Associations among mothers of other or unknown race or ethnicity were elevated in both tertiles, but not statistically significant.

**Table 3:** Odds ratios (OR)^a^ and 95% confidence intervals (95% CI) for the association between Index of Concentration at the Extremes (ICE)^b^ and stillbirth among mothers by racial and ethnic group, Harris County, TX, 2007–2020

		Non-Hispanic Black mothers		Non-Hispanic White mothers		Hispanic mothers		Mothers of other or unknown race or ethnicity
	*n*^c^	OR (95% CI)	*n*^c^	OR (95% CI)	*n*^c^	OR (95% CI)	*n*^c^	OR (95% CI)
**ICE**								
Tertile 1	474	Ref	49	Ref	621	Ref	26	Ref
Tertile 2	357	0.95 (0.83, 1.10)	79	0.85 (0.60, 1.22)	625	1.06 (0.94, 1.19)	53	1.39 (0.85, 2.27)
Tertile 3	436	1.03 (0.90, 1.18)	589	0.82 (0.60, 1.12)	738	1.01 (0.90, 1.14)	152	1.26 (0.80, 1.98)

^a^Models adjusted for maternal age, education, smoking during pregnancy, and pre-pregnancy BMI

^b^Contrasting high-income non-Hispanic White persons with low-income Persons of Color; tertile 1, most deprived; tertile 3, most privileged

^c^No. of stillbirths

Because of the notable racial and ethnic disparities in stillbirth between non-Hispanic Black and non-Hispanic White mothers (see Table 2), we conducted Blinder-Oaxaca Decomposition analysis for these two groups. Here, the predicted difference in the probability of stillbirth for non-Hispanic Black and non-Hispanic White mothers was 0.0042 (95% CI 0.0037–0.0047) (Table 4). Together, the predictors in the model explained 21.2% of the Black-White differences in the prevalence of stillbirth. Individually, the relative contributions to the disparity gap were largest for mothers with the lowest levels of education (high school or less) (12.3%), followed by mothers living in the most privileged neighborhoods (ICE tertile 3) (9.7%) and obese mothers (7.2%). In contrast, age contributed negatively, suggesting that the disparity gap would widen even further if the age distribution of non-Hispanic Black mothers was like that of non-Hispanic White mothers (i.e., if it was shifted towards older ages). Differences in smoking between the two groups had a negligible effect (− 0.7%), likely due to the low prevalence (< 5%) of this risk factor in both non-Hispanic White and non-Hispanic Black mothers.

**Table 4:** Results of the Oaxaca-Blinder decomposition of the disparity in stillbirth between non-Hispanic Black and non-Hispanic White mothers, Harris County, TX, 2007–2020

	Estimate (95% CI)	
Predicted probability		
Non-Hispanic Black mothers	0.0076 (0.0072, 0.0081)	
Non-Hispanic White mothers	0.0035 (0.0032, 0.0037)	
Black-White disparity	0.0042 (0.0037, 0.0047)	
Explained difference	0.0009 (0.0003, 0.0014)	
Total difference explained (%)	21.23%	

Predictors	Coefficient	% difference explained
Mother’s age (y)		
< 20	− 0.000002	− 0.05%
20–24	− 0.000083	− 2.0%
25–29	Ref	
30–34^a^	− 0.0001026	− 2.5%
≥ 35^a^	− 0.0001198	− 2.9%
Sum		** − 7.4%**
Mother’s education		
High school or less^a^	0.000513	12.3%
Some college^a^	0.0000765	1.8%
Bachelor’s degree or more	Ref	
Sum		**14.1%**
Pre-pregnancy BMI		
Underweight	− 0.00000215	− 0.1%
Normal weight	Ref	
Overweight^a^	0.0000562	1.3%
Obese^a^	0.0003008	7.2%
Sum		**8.5%**
Smoking during pregnancy		
No	Ref	
Yes^a^	− 0.0000305	** − 0.7%**
ICE^b^		
Tertile 1	Ref	
Tertile 2	− 0.0001246	− 3.0%
Tertile 3	0.0004029	9.7%
Sum		**6.7%**

^a^*p* < 0.05

^b^Index of Concentration at the Extremes, contrasting high-income non-Hispanic White persons with low-income Persons of Color; tertile 1, most deprived; tertile 3, most privileged

## Discussion

As reported previously for US mothers nationwide [2], a major disparity exists in stillbirth among non-Hispanic Black mothers as compared to non-Hispanic White mothers who live in the greater Houston area. Consistent with the Hispanic paradox (or more generally the immigrant paradox) [29], the prevalence of stillbirth among Hispanic mothers (and mothers of unknown or other races and ethnicity) was closer to that of non-Hispanic White mothers. In our primary analyses, we found differences in the impact of racialized economic segregation among mothers of different racial and ethnic groups. Additionally, to our knowledge, we are the first investigation to explore the contribution of segregation to Black-White disparities in stillbirth; we found that ICE, along with age, smoking, education, and pre-pregnancy BMI, explained 21.2% of the disparity between these two groups of mothers.

While our findings did not achieve statistical significance, there was a reduction in the odds of stillbirth for non-Hispanic White mothers living in the most privileged neighborhoods as compared to those living in deprived neighborhoods. In contrast, our results for non-Hispanic Black mothers, Hispanic mothers, and mothers with unknown or other race or ethnicity suggest that residence in more privileged neighborhoods did not confer the same benefits and may even increase risks of stillbirth. While comparisons to previous studies are not straightforward because of the different metrics to evaluate segregation, our results are consistent for non-Hispanic White but not non-Hispanic Black mothers. In two early investigations among mothers in New York City, increased rates of stillbirth were reported among both Black and White mothers who lived in areas with a relatively greater number of Black than White births [15, 16]. A later study in Georgia reported higher odds of stillbirth among Black mothers living in counties with the highest levels of Black-White segregation, whereas lower odds were found for non-Hispanic White mothers [17]. In another study limited to hospital reference regions in the USA, the odds of stillbirth were lower among non-Hispanic Black mothers living in less segregated regions; patterns, while similar, were not as strong for non-Hispanic White mothers [18].

Previous studies suggest several reasons for the differential impact of segregation on maternal and child health outcomes by race and ethnicity. For example, historical segregation and disinvestment have been observed in neighborhoods with high concentrations of racial and ethnic groups, leading to fewer high-quality obstetric care facilities and fewer maternal health resources [29, 30]. Segregation additionally results in differential access to other resources due to institutional racism, discrimination, and implicit bias in healthcare settings [31]. Moreover, the compounding effects of race, socioeconomic status, and gender, which can lead to a higher prevalence and poorer management of chronic diseases among certain subgroups and greater exposures to psychosocial stressors, have also been suggested to contribute to the differential effects of the neighborhood on adverse health outcomes among racial and ethnic groups [30].

Given our findings, it is possible that non-White mothers living in relatively privileged neighborhoods, in which they are the minority, might experience discrimination that offsets the protective effects associated with economic privilege. It is well documented that the chronic stress associated with experiences of racial discrimination and stigma [32, 33], as well as the potentially restricted support network to cope with these stressors [34], has an effect on adverse birth outcomes for Black mothers [35]. Fewer studies have included Hispanic mothers in their investigations, as evidenced by a review of the literature on the role of discrimination in increasing risks of adverse birth outcomes [36], although there is recent evidence that it impacts perinatal health in this population as well [37]. While the underlying biological mechanisms are not fully understood, it has been hypothesized that repeated exposure to stressful conditions may result in increased allostatic load or cumulative “wear and tear” on the body due to physiological manifestations of chronic stress. One such manifestation is activation of the hypothalamic–pituitary–adrenal [HPA] axis, which in turn can lead to inhibition of the hypothalamic-pituitary-ovarian (HPO) axis and result in a cascade of endocrine, paracrine, and neural events that can adversely impact pregnancy [38]. A second mechanism through which stress may impact perinatal health is through elevated oxidative stress, possibly resulting in abnormal placentation and/or placental dysfunction that may contribute to adverse pregnancy outcomes including fetal death [39].

Our null findings for the association between living in privileged neighborhoods and stillbirth for non-Hispanic Black and Hispanic mothers (and the suggested finding of elevated risks for mothers whose race and ethnicity was unknown or classified as other) may have non-causal explanations, as might arise if mothers who are highly susceptible to having a stillbirth experience early pregnancy loss [40]. Under this scenario of live birth bias, exposure to extreme deprivation could differentially result in early losses, so that non-White mothers living in deprived neighborhoods would appear to have a lower prevalence of stillbirth. Further, there may also be unmeasured risk factors of both early pregnancy loss and stillbirth, such as inadequate access to prenatal care, or the potential weathering effect of deprivation or racial discrimination [41]. In this case, conditioning on survival past early pregnancy would create a non-causal path between social polarization and stillbirth and introduce collider stratification bias, leading to specious associations [40, 42].

In our study, we also set out to explore the relative contribution of segregation to racial disparities in stillbirth [43] and found in our decomposition analysis that living in the most deprived neighborhoods explained a modest proportion (9.7%) of the disparity between non-Hispanic Black and non-Hispanic White mothers. While no previous investigation has examined this question as it relates to stillbirth, several studies have evaluated the role of segregation in explaining disparities in other birth outcomes (although none of these applied Blinder-Oaxaca decomposition methods). In a two-stage multi-level analysis of singleton births nationwide in the USA, for example, county-level measures of segregation modified associations between maternal race and ethnicity and preterm birth and low birth weight [11]. In another investigation, Wallace et al. applied a series of Poisson regression models and reported that accounting for ICE reduced the wide disparity in risk of infant mortality between non-Hispanic Black and non-Hispanic White mothers living in Detroit, Michigan, whereas the addition of individual-level covariates had little effect [44].

Our study had several strengths and weaknesses. One strength included the use of a large, racially and ethnically, and socioeconomically diverse cohort, which allowed us to examine race- and ethnicity-specific associations of the impact of ICE on stillbirth risk, including Hispanic mothers and mothers of other or unknown race and ethnicity. Even so, our results were relatively imprecise, pointing to the difficulty in studying a rare outcome. Likewise, although our data covered an extended (14-year) period, we were also not powered to evaluate temporal trends in associations of segregation and stillbirth, an analysis that could be undertaken in the future to explore potential influences of changes in socioeconomic factors, demographic shifts, or policy. Another limitation was the lack of information on fetal death certificates on socioeconomic status (SES) beyond maternal education (i.e., information on income or payor status was not available), and this may have resulted in residual confounding. We also did not have information on residential history during pregnancy; however, a prior study in Texas reported that moves during pregnancy, when they occurred, were not very far [45]; further, any exposure misclassification, if present, is expected to have biased effect estimates toward the null. Another strength of our study was the use of a multi-dimensional metric (ICE) that characterizes relative differences between privilege and deprivation, accounting both for race and ethnicity and income [46], and the construction of this metric was based on the income distribution in the study area (rather than reliance on distributions for the US population as a whole that has been criticized as not being representative of the target population) [47]. ICE offers another advantage in its ability to capture greater variation at the census tract level compared to traditional metrics that may have limited utility at small geographic scales [20]. Notwithstanding these advantages, we recognize a limitation in that census tracts may not be optimal representations of the neighborhoods in which people live.

Our study of a large, diverse urban area reveals persistent racial disparities in stillbirth risk, with a complex relationship with neighborhood segregation across racial and ethnic groups. Our findings indicate that living in more privileged neighborhoods potentially benefits White mothers but shows no clear advantage for mothers in other racial and ethnic groups. While racialized economic segregation, as measured by ICE, was a contributor to Black-White disparities in stillbirth, along with education and pre-pregnancy BMI, much of the disparity gap could not be explained with the predictors that were examined in the decomposition analysis. These results highlight the need for more nuanced investigations across diverse geographic and demographic contexts, focused on examining the intersectionality of individual social identities and experiences that may lead to chronic stress and other contextual factors (e.g., inequalities in the criminal justice or healthcare systems) on increased risks for stillbirth. Additionally, evaluating policy interventions is also needed. This work is essential for reducing health disparities and ensuring that all mothers have the best possible opportunity for a healthy pregnancy outcome.

## Data Availability

The data used in this study were obtained from the Texas Department of State Health Services (DSHS) with approval from the TX DSHS Institutional Review Board (IRB). These data are not publicly available due to legal and ethical restrictions.
